# Single Cell RNA-seq Data Analysis Reveals the Potential Risk of SARS-CoV-2 Infection Among Different Respiratory System Conditions

**DOI:** 10.3389/fgene.2020.00942

**Published:** 2020-08-20

**Authors:** Qiang Zhang, Yuanyi Yue, Huiwen Tan, Yishu Liu, Yin Zeng, Li Xiao

**Affiliations:** ^1^Department of Pulmonary and Critical Care Medicine, Shengjing Hospital of China Medical University, Shenyang, China; ^2^Department of Gastroenterology Medicine, Shengjing Hospital of China Medical University, Shenyang, China; ^3^Sleep Medicine Center, Shengjing Hospital of China Medical University, Shenyang, China

**Keywords:** *ACE2*, *TMPRSS2*, COVID-19, respiratory system, single cell transcriptome

## Abstract

COVID-19 (Coronavirus Disease 2019) has been an ongoing pandemic, resulting in an increase in people being infected globally. Understanding the potential risk of infection for people under different respiratory system conditions is important and will help prevent disease spreading. We explored and collected five published and one unpublished single-cell respiratory system tissue transcriptome datasets, including idiopathic pulmonary fibrosis (IPF), aging lungs (mouse origin data), lung cancers, and smoked branchial epithelium, for specifically reanalyzing the *ACE2* and *TMPRSS2* expression profiles. Compared to normal people, we found that smoking and lung cancer increase the risk for COVID-19 infection due to a higher expression of *ACE2* and *TMPRSS2* in lung cells. Aged lung does not show increased risk for infection. IPF patients may have a lower risk for original COVID-19 infection due to lower expression in AT2 cells but may have a higher risk for severity due to a broader expression spectrum of *TMPRSS2*. Further investigation and validation on these cell types are required. Nonetheless, this is the first report to predict the risk and potential severity for COVID-19 infection for people with different respiratory system conditions. Our analysis is the first systematic description and analysis to illustrate how the underlying respiratory system conditions contribute to a higher infection risk.

## Introduction

COVID-19 (Coronavirus Disease 2019), which is caused by severe acute respiratory syndrome coronavirus 2 (SARS-CoV-2), has been recognized as a global public health crisis, infecting more than more than 1 million people and causing more than 50,000 deaths (Early April, WHO statistic). With the number of infections, death, and infected countries climbing even higher, the World Health Organization (WHO) has already declared the rapidly COVID-19 outbreak a pandemic. To date, data have shown that older age and comorbidity such as hypertension (most common), diabetes, and coronary heart diseases are highly related to the in-hospital death rate (Huang et al., 2020; Zhou F. et al., 2020). Estimated mortality following COVID-19 infection is considered a rate of 5.7% (5.5–5.9) (Baud et al., 2020). Currently, although there exists a supporting treatment guideline, still no specific medicine is proved to effectively prevent or treat COVID-19, according to WHO. Therefore, understanding which population is under higher risk is a crucial task to prevent disease spreading and decrease the mortality.

The novel coronavirus (2019-nCoV, SARS-CoV-2) is considered as a member of seven known coronaviruses that could infect humans. Although whether there exists an intermediate host of this virus is still under investigation, the mechanism of how this virus enters human cells is much clearer. As both 2019-nCoV and SARS-CoV share an ancestor which resembles the bat coronavirus HKU9-1, the similar spike protein 3-D structures are all considered to bind strongly to the angiotensin-converting enzyme 2 (*ACE2*) for entering the human cells (Xu X. et al., 2020). Cells with a high *ACE2* expression may be more susceptible to the infection as target cells, which include lung type II alveolar cells (AT2) (Zhou P. et al., 2020). More recently, Hoffmann et al. (2020) also reported a serine protease named *TMPRSS2*, which is also crucial for the virus to enter the human cells and S protein priming. Both *ACE2* and *TMPRSS2* were reported to be expressed in bronchial transient secretory cells (Lukassen et al., 2020). Interestingly, *TMPRSS2*-expressing cells have also been demonstrated to enhance the SARS-CoV-2 infection (Matsuyama et al., 2020). These studies suggest that *ACE2* and *TMPRSS2* may play a central role in SARS-CoV-2 infection.

Single-cell transcriptome analysis provides a higher resolution of cellular differences and generates a better understanding of the function of an individual cell, in the context of its microenvironment (Eberwine et al., 2014). Recently, a single-cell-based data-mining research demonstrated that *ACE2* is not only expressed in lung AT2 cells but also highly expressed in other tissues, including kidney, myocardial cells, testicle, and bladder, resulting in potential infection or damage in these organs (Zou et al., 2020). Indeed, a recent report demonstrated that COVID-19 could cause acute myocardial injury (AMI) (Xu H. et al., 2020). Kidney functions were also reported to be severely impaired in many patients with SARS-CoV-2 infection (Li et al., 2020). A research group in Italy also reported that *ACE2* and *TMPRSS2* variants and expression could contribute to the different severities of COVID-19 (Asselta et al., 2020). These studies suggest that using *ACE2* and related protein expression profiles to demonstrate an infectable organ risk map is significant.

Although clinical studies have proved that people with older ages or certain underlying conditions are at higher risk for COVID-19 illness, in fact, there is little evidence focusing on how the patient conditions deteriorate with these risk factors. In this study, we focused on different respiratory system conditions especially. We explored five publicly available single-cell respiratory system tissue transcriptome datasets (GSE122960, GSE124872, GSE127465, GSE131391, and Adams dataset which is only available for interactive viewing) that focused on different conditions, including idiopathic pulmonary fibrosis (IPF), aging lungs (mouse origin data), lung cancers, and smoked branchial epithelium, for specifically reanalyzing the *ACE2* and *TMPRSS2* expression profiles (Adams et al., 2019; Angelidis et al., 2019; Duclos et al., 2019; Reyfman et al., 2019; Zilionis et al., 2019). Compared to the normal lungs, we found the following: (1) IPF patients may have a lower risk for COVID-19 infection due to a lower expression in AT2 cells but may have a higher risk for severity due to a broader expression spectrum of *TMPRSS2*; (2) The infection risk for smoking people is slightly higher than for non-smokers due to a higher expression of *TMPRSS2*; (3) Reanalyzing mouse lung tissue data shows that aged lung does not have a higher risk for infection due to the non-significant change of *ACE2* and *TMPRSS2* expression; (4) Lung cancer could potentially produce novel cell types with a high *TMPRSS2* expression, which might increase the risk of COVID-19 infection. Our data shed a light on understanding the risk of COVID-19 infection among people with different respiratory system conditions. COVID-19 (Coronavirus Disease 2019), which is caused by severe acute respiratory syndrome coronavirus 2 (SARS-CoV-2), has been recognized as a global public health crisis, infecting more than 1 million people and causing more than 50,000 deaths (Early April, WHO statistic). With the number of infections, death, and infected countries climbing even higher, the World Health Organization (WHO) has already declared the rapidly COVID-19 outbreak a pandemic. To date, data have shown that older age and comorbidity such as hypertension (most common), diabetes, and coronary heart diseases are highly related to the in-hospital death rate (Huang et al., 2020; Zhou F. et al., 2020). Estimated mortality following COVID-19 infection is considered a rate of 5.7% (5.5–5.9) (Baud et al., 2020). Currently, although there exists a supporting treatment guideline, still no specific medicine is proved to effectively prevent or treat COVID-19, according to WHO. Therefore, understanding which population is under higher risk is a crucial task to prevent disease spreading and decrease the mortality.

## Materials and Methods

### Data Resources

Raw read count matrices are downloaded from GEO (GSE122960 for Reyfman dataset, GSE131391 for Duclos dataset, GSE124872 for Angelidis dataset) (Adams et al., 2019; Angelidis et al., 2019; Duclos et al., 2019; Reyfman et al., 2019; Zilionis et al., 2019). Author-normalized expression matrices are downloaded from GEO (GSE127465 for Zilionis dataset) (Zilionis et al., 2019). Online visualization was performed for the Adams dataset from http://www.ipfcellatlas.com/due to unavailability of raw data (Adams et al., 2019). GTEx TPM normalized data was downloaded from the GTEx website: https://www.gtexportal.org/home/datasets. The GTEx sample annotation was downloaded from https://gtexportal.org/home/histologyPage.

### Single-Cell RNA-Seq Data Analysis

For Reyfman dataset, Duclos dataset, and Angelidis dataset, single-cell RNA-seq data was mainly analyzed using a standard Seurat V3 (Butler et al., 2018) workflow as described below. Genes which were detected in less than 1% of total cells and cells which express less than 500 genes were filtered out before further processing. The expression matrices were then normalized by TPM/10 and transformed by a natural logarithm (TPM for each gene in each cell was calculated by multiplying the proportion of the transcripts of that gene in the cell by 1,000,000). The top 2000 variable genes were selected by the Seurat implemented method “FindVariableFeatures” using the “vst” method. The matrices were then reduced to those 2000 rows for integration, dimension reduction, and clustering. Integration was performed with the anchor method in Seurat V3. Dimension reduction was first performed with PCA and then with UMAP using top 20 PCA projections as input. Nearest neighbors were defined among cells with the KNN method (FindNeighbors in Seurat), and cells were then grouped with Louvain algorithm (FindClusters in Seurat). For downstream analysis, differential expression analysis was performed with the MAST method (Finak et al., 2015), which uses a hurdle model to tackle the high dropout effects in single-cell data.

For the Zilionis dataset, the cell clusters and annotations were directly adapted from the authors’ metadata due to heterogeneity among sample batches. Visualization of this dataset was performed with Seurat.

### Bulk RNA-Seq Data Analysis

The GTEx data was visualized using ggplot2 R package. Statistical test among groups was performed using one-way ANOVA, and paired comparison was done with Tukey HSD.

## Results

### The Expression of *ACE2* and *TMPRSS2* in Lung Cells With Fibrosis

**FIGURE 1:**
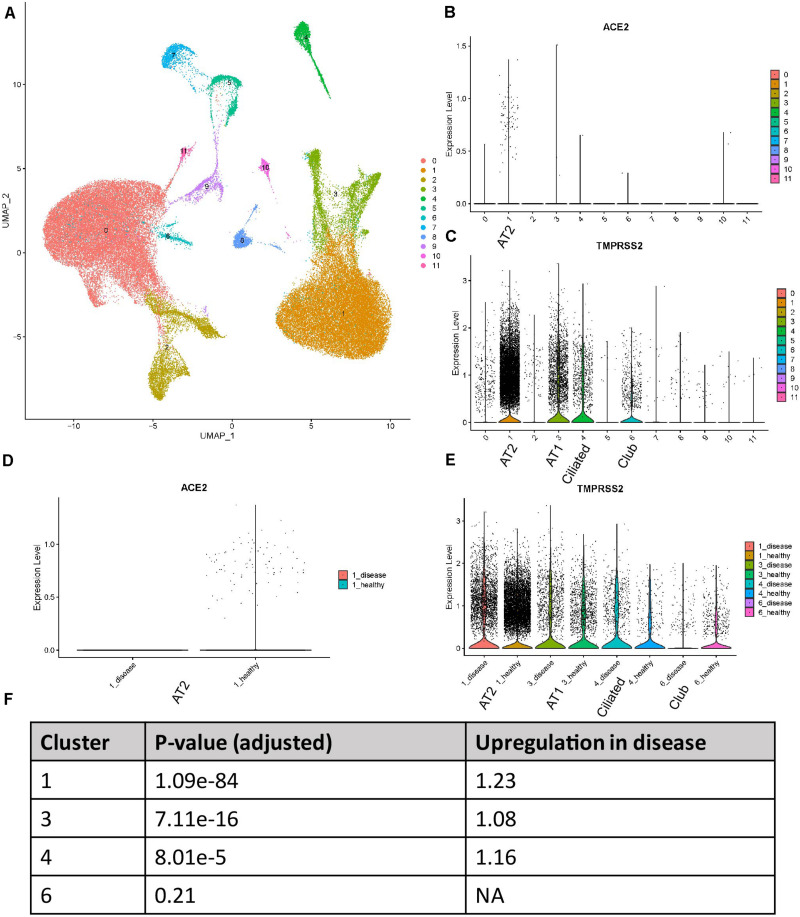
Expression profiles of ACE2 and TMPRSS2 in IPF lung tissues (Reyfman dataset) **(A)** UMAP visualization of 12 cell clusters. **(B)** Expression profile of ACE2 in the cell clusters. **(C)** Expression profile of TMPRSS2 in the cell clusters. **(D)** Comparison of ACE2 expression between IPF and healthy groups in cluster 1. **(E)** Comparison of TMPRSS2 expression between IPF and healthy groups in clusters 1, 3, 4, and 6. **(F)** *P*-values and fold changes of TMPRSS2 in different clusters of IPF conditions compared with healthy conditions.

We reanalyzed the dataset reported by Reyfman et al. (2019). This dataset includes tissues from eight healthy donors and eight patient samples. 77275 cells passed the quality control and were used for the downstream analysis. The cells formed 12 individual clusters (Figure 1A). The expression profiles of *ACE2* and *TMPRSS2* were checked and visualized with violin plots (Figures 1B,C). It could be clearly seen that *ACE2* was almost solely expressed in cluster 1 while *TMPRSS2* was expressed in clusters 1, 3, 4, and 6. Cell type markers reported from the original paper were used to annotate those clusters (Supplementary Figure 1). *SFTPC*, *AGER*, *TPPP3*, and *SCGB3A2* were used to annotate clusters 1, 3, 4, and 6, respectively, to AT2, AT1, ciliated cells, and club cells. It is worth mentioning that *SCGB3A2* was not exclusively for club cells, and it is consistent with the original paper. Thus, we conclude that *ACE2* was highly expressed in AT2 cells while *TMPRSS2* showed an expression in not only AT2 cells but also AT1, club cells, and ciliated cells.

We then compared the expression level of these two genes between healthy and disease conditions. To utilize the power of single-cell RNA-seq for “*in silico* sorting,” we performed the comparison within each individual cluster, not averaging all cells among clusters. To tackle the dropout effects of single-cell data, we used the MAST method which applied a hurdle model. For *ACE2*, interestingly, all the counts for this gene in this dataset were contributed by healthy tissues (Figure 1D). This was not too surprising due to the overall low expression of this gene. For *TMPRSS2*, in clusters 1, 3, and 4, the disease group showed an elevated expression compared with the healthy group (Figure 1E). *P*-values and fold changes were also calculated (Figure 1F). In summary, *ACE2* demonstrated a higher expression in healthy AT2 cells compared with those with IPF, while *TMPRSS2* showed an opposite trend in AT2, AT1, and ciliated cells.

**FIGURE 2:**
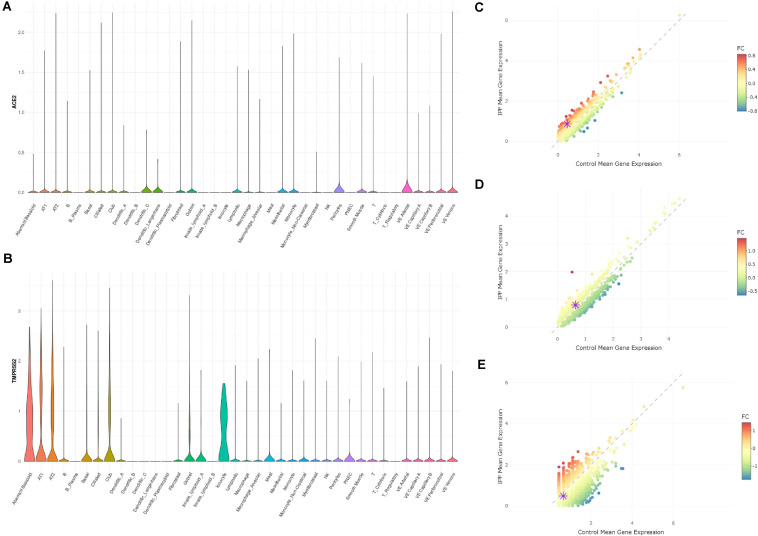
Expression profiles of ACE2 and TMPRSS2 in IPF lung tissues (Adams dataset) **(A)** Expression profile of ACE2 in the cell types annotated by authors. **(B)** Expression profile of TMPRSS2 in the cell types annotated by authors. **(C)** Comparison of TMPRSS2 expression between IPF and healthy groups in cell type AT1 (upregulated in IPF by 1.89-fold). **(D)** Comparison of TMPRSS2 expression between IPF and healthy groups in cell type AT2 (upregulated in IPF by 1.23-fold). **(E)** Comparison of TMPRSS2 expression between IPF and healthy groups in cell type ionocytes (downregulated in IPF by 0.72-fold).

A new dataset was reported with a preprint by Adams et al., including 312928 cells from lung tissues of healthy people and patients with lung fibrosis (Adams et al., 2019). Due to the availability of the data, we were not able to reanalyze the data. However, through the online tool kindly provided by authors^1^, we could visualize the expression profiles of *ACE2* and *TMPRSS2* in this dataset. In this dataset, *ACE2* showed an expression not restricted to AT2 cells, but also in other lung cell types including AT1, basal, ciliated, and club cells (Figure 2A). We reason that the expression level of *ACE2* was quite low and thus cannot be detected by relatively moderate-sized datasets. With 0.3 million cells, *ACE2* showed and expanded expression spectra. In all the cell types, *ACE2* did not show expression differences between disease and control samples. As for *TMPRSS2*, it showed an expression in AT1, AT2, basal, ciliated, club, and ionocyte cells, which is largely consistent with our findings in the Reyfman dataset. In AT1 (Figure 2C) and AT2 (Figure 2D) cells, the *TMPRSS2* expression was higher in IPF tissues compared with control (1.89-fold for AT1 and 1.23-fold for AT2), while lower in ionocytes (0.72-fold). This also showed consistency with findings from the Reyfman dataset. It is also worth mentioning that in the IPF lung, the authors discovered a novel cell type named “Aberrant basaloid.” Both *ACE2* and *TMPRSS2* showed an expression in this cell type (Figures 2A,B). This is an indication that IPF patients may have more cell types available for the virus infection.

### The Expression of *ACE2* and *TMPRSS2* in Human Bronchial Epithelium With Smoking Conditions

**FIGURE 3:**
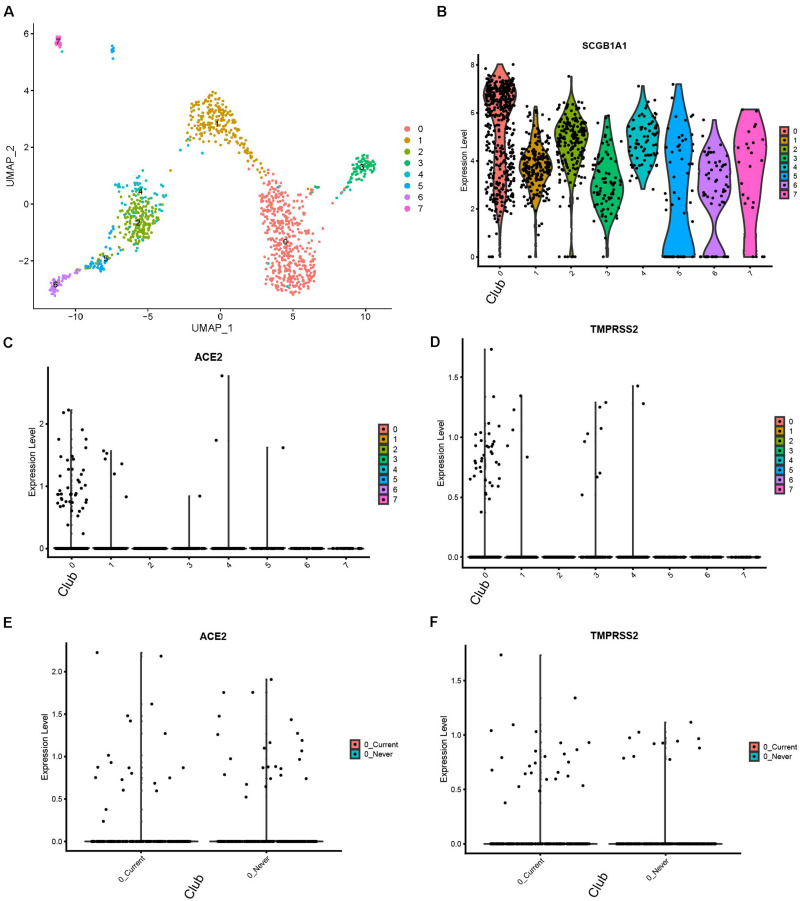
Expression profiles of ACE2 and TMPRSS2 in human bronchial tissues with smoking conditions (Duclos dataset) **(A)** UMAP visualization of eight cell clusters. **(B)** Cluster 0 highly express SCGB1A1, a club cell marker. **(C)** Expression profile of ACE2 in the cell clusters. **(D)** Expression profile of TMPRSS2 in the cell clusters. **(E)** Comparison of ACE2 expression in club cells between current smokers and never smokers. **(F)** Comparison of TMPRSS2.

We reanalyzed the dataset reported by Duclos et al. (2019). This dataset included samples from 12 donors, among which six were current smokers and the rest were never smokers. The cells formed eight clusters (Figure 3A). The expression profiles of *ACE2* and *TMPRSS2* were checked and visualized with violin plots (Figures 3C,D). It could be seen that both *ACE2* and *TMPRSS2* were majorly expressed in cluster 0. We used originally reported markers to annotate this cluster, and with the marker *SCGB1A1*, it was annotated as club cells (Figure 3B). We then compared the expression level of these two genes between current smokers and never smokers within cluster 0. For *ACE2*, there appeared no differences between two groups (*p*-value 0.9122). For *TMPRSS2*, the current smoker group showed a slightly higher expression (1.08-fold, *p*-value 0.00519). Thus, we conclude that club cells are the major potential targets for the COVID-19 virus in the human bronchial epithelium, and the risk for smoking people is slightly higher than non-smokers.

### The Expression of *Ace2* and *Tmprss2* in Mouse Lung With Different Ages

**FIGURE 4:**
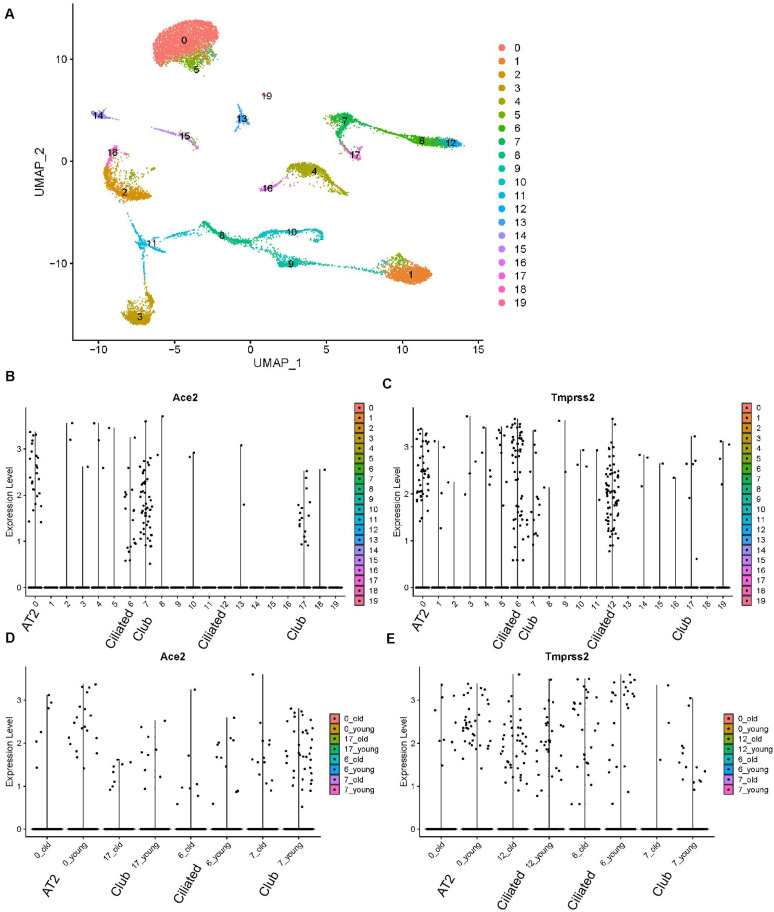
Expression profiles of Ace2 and Tmprss2 in young and aged mouse lung tissues (Angelidis dataset). **(A)** UMAP visualization of 20 cell clusters. **(B)** Expression profile of Ace2 in the cell clusters. **(C)** Expression profile of Tmprss2 in the cell clusters. **(D)** Comparison of Ace2 expression between young and aged groups in clusters 0, 6, 7, and 17. **(E)** Comparison of Tmprss2 expression between young and aged groups in cluster 0, 6, 7, and 12.

We reanalyzed the dataset reported by Angelidis et al. (2019). This dataset included samples from 8.3-month-old mice and 7.24-month-old mice, which were defined as young and old mice, respectively. The cells formed 20 clusters (Figure 4A). The expression profiles of *Ace2* and *Tmprss2* were checked and visualized with violin plots (Figures 3B,C). *Ace2* and *Tmprss2* showed quite similar expression profiles in the mouse tissue, with *Ace2* majorly in clusters 0, 6, 7, and 17, while *Trpmss2* were majorly in clusters 0, 6, 7, and 12. We used originally reported markers to annotate these clusters. With *Sftpd*, *Scgb1a1*, and *Foxj1*, we annotated cluster 0 to AT2 cells, cluster 6/12 to ciliated cells, and cluster 7/17 to club cells, respectively (Supplementary Figure 2). We then compared the expression levels of these two genes between young and old mice within these clusters. For all the eight comparison groups (two genes, four groups each), none of the genes and clusters showed any differences between young and old mice (*p*-value ranging from 0.2770 to 0.8571) (Figures 4D,E). In summary, we found that the expression profiles of *Ace2* and *Tmprss2* were quite similar between mouse lungs and human lungs, for they were both expressed in AT2, ciliated, and club cells. With the mouse study, we conclude that aged lung does not express more receptors for the virus infection than young ones.

In order to assess our conclusions in the context of human lung tissues, given that there are no available single-cell human lung data with different ages (and with multiple samples), we analyzed bulk RNA-seq data from 577 human lung tissues, obtained from the GTEx platform^2^. There are six age groups among the data, from 20 to 79 years of age. We visualized the expression (TPM) of *ACE2* and *TMPRSS2* among those age groups and observed no obvious differences among ages. We performed one-way ANOVA test for both genes among ages and Tukey test for paired comparison. For both genes, none of the age groups showed differential expression with each other (Supplementary Figure 3). Thus, with the human bulk RNA-seq data, we draw the same conclusion with our findings in mouse data. We conclude that the overall expression of ACE2 and TMPRSS2 does not change with age in the human lung tissue.

### The Expression of *ACE2* and *TMPRSS2* in Non-Small-Cell Lung Cancers (NSCLC)

**FIGURE 5:**
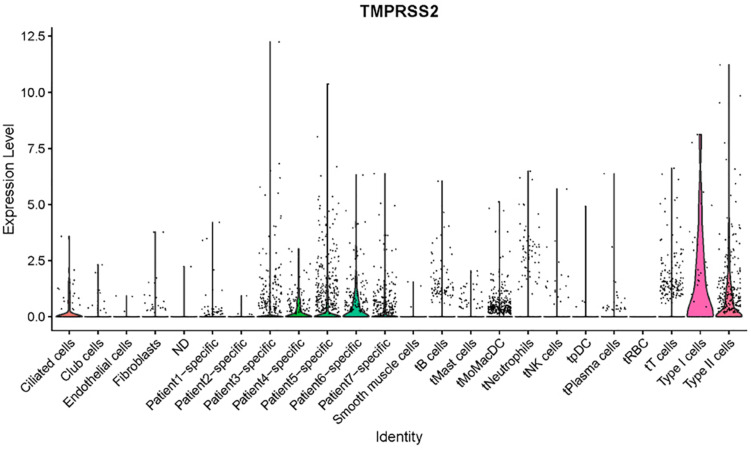
Expression profiles of TMPRSS2 in NSCLC tumor tissues (Zilionis dataset).

We checked a single-cell dataset reported by Zilionis et al. for *ACE2* and *TMPRSS2* expression in human NSCLC tissues (Zilionis et al., 2019). Due to the heterogeneity of patients reported in the original study, we tend not to annotate the cells by ourselves, while instead, we used the metadata provided by the authors including cell annotation. We then tried to visualize the expression for the two genes of interest. *ACE2* was not included in the expression matrix, which is possibly because it got filtered out due to low expression level. *TMPRSS2* showed a high expression level in AT1 and AT2 cells, with some expression in ciliated cells, which had consistency with our findings in other datasets (Figure 5). Interestingly, the authors annotated a few cells as “patient-specific” cells in this dataset. They could not be annotated with conventional markers and were likely to be novel types in the condition of cancer. In the patient-specific cells for patients 4, 5, and 6, the expression of *TMPRSS2* seemed higher than the ones of patients 1, 2, 3, and 7. This result indicates that lung cancer could potentially produce novel cell types with *TMPRSS2* expression, which might increase the risk of COVID-19 infection. This of course, remains a rough prediction given that we do not know the expression of ACE2 in those cell types yet.

## Discussion

COVID-19 has been a global pandemic, infecting millions of people. Preventing the disease from spreading is currently the most urgent thing for all human beings. Herein, we demonstrated a risk prediction for those people with different respiratory system conditions for COVID-19 infection. We focused on using single-cell-level *ACE2* and *TMPRSS2* expression data as a predictor, which has been validated to be meaningful at a certain degree by clinical data, showing that COVID-19 infects not only the lung but also other organs (Li et al., 2020; Zou et al., 2020). As COVID-19 infection is believed to cause more severe symptoms in those who already have underlying conditions or older ages, herein we proposed some of the potential reasons using integrated single-cell RNA-seq analysis. It is worth mentioning that some studies and recent preprints have also reported ACE2 expression profiles in the human lung (Uhal et al., 2013; Chow and Chen, 2020), while our study aims at having a more systematic assessment that includes not only ACE2 but also TMPRSS2, a newly confirmed protein required for COVID-19 entry.

We demonstrated that people with smoking habits tend to have a higher risk for COVID-19 infection. We also speculate that a more severe symptom may be developed in these populations. It is worth mentioning that for cells of smoking people, we only observed an upregulation of TMPRSS2 while the level of ACE2 did not change. Given that the dynamics of the virus infection have not been fully revealed, it is still uncertain to what extent a high level of TMPRSS2 alone, without the change of ACE2, would lead to a higher infection efficiency. For people with non-small-cell lung cancers, very interestingly, some of the patient-specific cell groups, which might be from the tumor heterogeneity, showed considerable expression of TMPRSS2, with the ACE2 level unknown due to the prefiltering of the data. Without knowing the expression of ACE2, we cannot gain a conclusion on whether the patient specific cells are potential targets for the COVID-19.

For the aged lung, although our single-cell analysis is based on a mouse dataset, our data strongly supports that aging may not be a direct factor for contributing to infection risk. The bulk RNA-seq data from humans also supported this conclusion. We also noticed another preprint manuscript (Booeshaghi and Pachter, 2020) which drew a seemingly different conclusion from our analysis in the expression of gene ACE2. However, certain differences do exist in the analysis from the two groups. First, the major conclusion in our study only focuses on the Ace2 expression within three cell types, which do not consider any other cell types in the data, while the other manuscript evaluated the proportion of Ace2-expressing cells in all cell types and found that younger mice had a higher overall proportion. Second, the reason we chose the presented “within-cell-type” comparison is that the proportion of each cell type is likely to alter between different groups (age groups in this case) and would affect the assessment of gene expression level. For lowly expressed genes, the detection rate of the gene might be affected by the sampling (total cell numbers). Third, single-cell transcriptomics suffer from “drop-out” effects, especially for the lowly expressed genes such as Ace2 in this case. Thus, to study the differential expression of genes among groups, certain methods which are specifically designed from single-cell data need to be used (MAST method here).

For these patients with IPF, the data we have here are controversial. We speculate that they may have a lower risk for infection, but a higher risk for disease severity, due to a lower *ACE2* but a higher *TMPRSS2* expression. In fact, a lower *ACE2* expression may lead to a lower sensitivity for the virus entrance. However, more investigations and studies need to be done to validate these single-cell RNA-seq results.

We analyzed the public single-cell transcriptome dataset to demonstrate the potential cause for the COVID-19 infection risk map of people with different respiratory system conditions; however, we understand that our results have certain limitations. First of all, we do not have any experiment-based validation for these specific *ACE2*- and *TMPRSS2*-expressed cell populations. Secondly, all the analyses included here are only focused on the transcriptome, not at the protein level. It has been shown that the mRNA level has a limited power of predicting the protein level, which is due to the comprehensive translation and posttranslation regulations. Thus, the interpretation of our findings should be limited to the transcriptomics level. Thirdly, although these two genes are highly involved in infection progress, the whole picture of how these viral-response signal cascade works is still unclear. There could exist other genes highly involved in disease progress, thus making our analysis and conclusion not comprehensive. Nonetheless, our data provided the first systematic analysis for COVID-19 infection risk of people with different respiratory system conditions.

## Data Availability Statement

The datasets (GSE122960 for Reyfman dataset, GSE131391 for Duclos dataset, GSE124872 for Angelidis dataset) for this study can be found in the GEO and in http://www.ipfcellatlas.com/.

## Author Contributions

QZ performed the models, analyzed the data, and wrote the manuscript. YY contributed with data analysis and writing of the manuscript. HT, YL, and YZ contributed with writing of the manuscript. LX designed the research study and analyzed and wrote the manuscript. All authors contributed to the article and approved the submitted version.

## Conflict of Interest

The authors declare that the research was conducted in the absence of any commercial or financial relationships that could be construed as a potential conflict of interest.

## Footnotes

1 http://ipfcellatlas.com/

2 https://www.gtexportal.org/home/datasets
