# Supplementary material for: Single Cell RNA-seq Data Analysis Reveals the Potential Risk of SARS-CoV-2 Infection Among Different Respiratory System Conditions
Source: Front Genet. 2020 Aug 20;11:942. doi: 10.3389/fgene.2020.00942 (PMC7468458; doi:10.3389/fgene.2020.00942)
Supplement: Supplementary file 1 [file Presentation_1.pdf]

Supplementary Material

1 Supplementary Figures

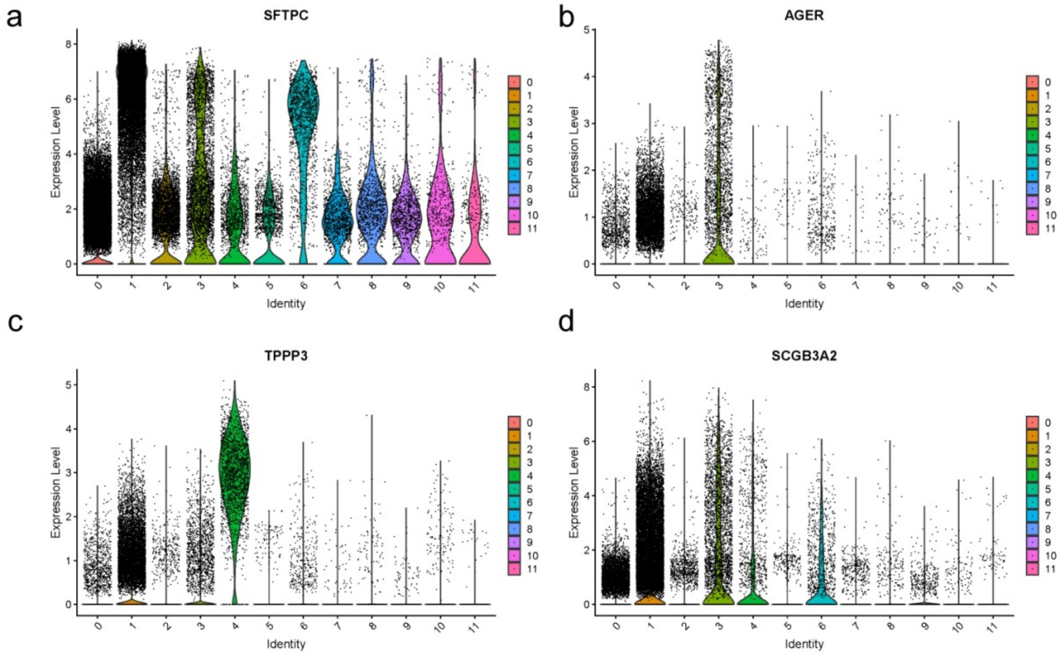

Supplementary Figure 1. The figure legends are required to have the same font as the main text, 12 point normal Times New Roman, single spaced. Please use a single paragraph for each legend and prepare the figures keeping in mind the PDF layout.

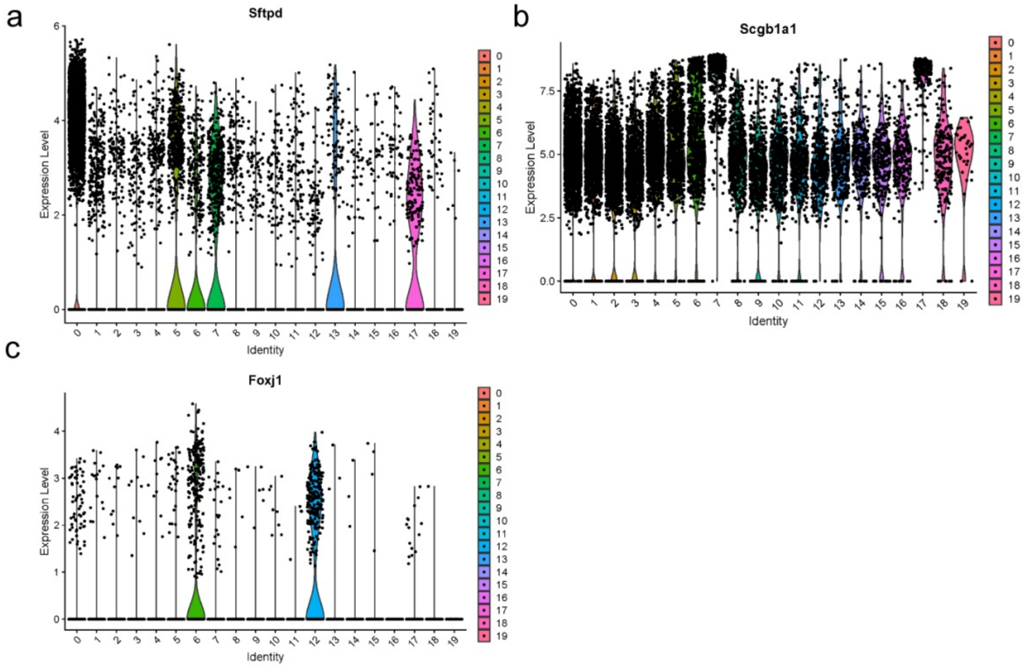

Supplementary Figure 2. Annotation of cell types with originally reported markers.

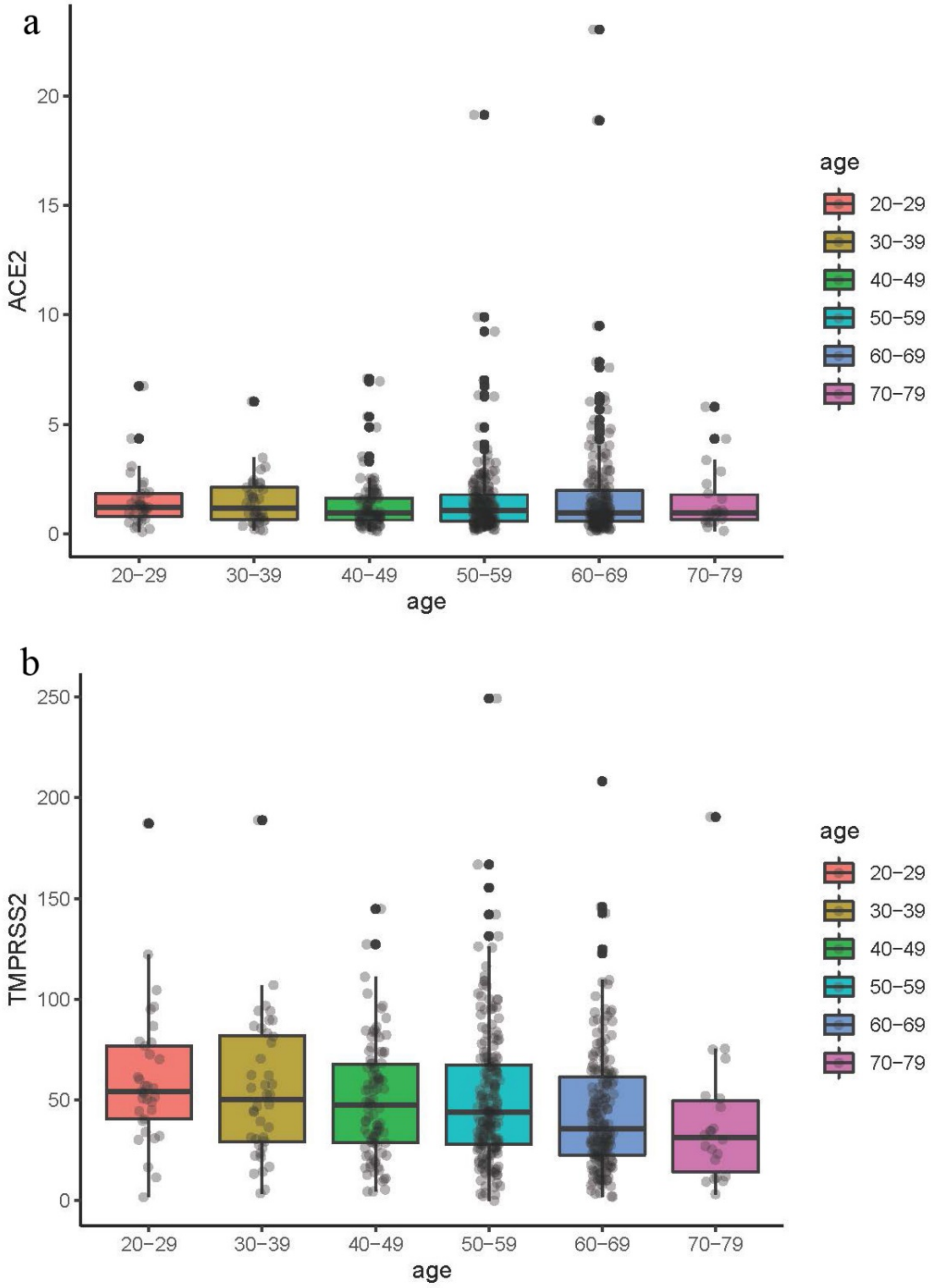

Supplementary Figure 3. (a) Expression of ACE2 in human lung samples (GTEx) with different age groups. The expression level is normalized with TPM. (b) Expression of TMPRSS2 in human lung samples (GTEx) with different age groups. The expression level is normalized with TPM.
